# Supplementary material for: Stabilization of three-dimensional charge order through interplanar orbital hybridization in PrxY1−xBa2Cu3O6+δ
Source: Nat Commun. 2022 Oct 19;13:6197. doi: 10.1038/s41467-022-33607-z (PMC9581994; doi:10.1038/s41467-022-33607-z)
Supplement: Supplementary file 1 — Supplementary Information [file 41467_2022_33607_MOESM1_ESM.pdf]

Supplementary Information for ***Stabilization of three-dimensional charge order through interplanar orbital hybridization in $Pr_xY_{1-x}Ba_2Cu_3O_{6+\delta}$***

Alejandro Ruiz,[1,*] Brandon Gunn,[1,*] Yi Lu,[2,3] Kalyan Sasmal,[1]
Camilla M. Moir,[1] Hai Huang,[4,†] Jun-Sik Lee,[4] Fanny Rodolakis,[5]
Timothy J. Boyle,[6,7] Morgan Walker,[6] Yu He,[8] Santiago Blanco-Canosa,[9,10]
Eduardo H. da Silva Neto,[6,7,11] M. Brian Maple,[1] and Alex Frano[1,12,‡]

[1]*Department of Physics, Center for Advanced Nanoscience,*
University of California, San Diego, California 92093, USA

[2]*National Laboratory of Solid State Microstructures and Department of Physics,*
Nanjing University, Nanjing 210093, China

[3]*Collaborative Innovation Center of Advanced Microstructures,*
Nanjing University, Nanjing 210093, China

[4]*Stanford Synchrotron Radiation Lightsource,*
SLAC National Accelerator Laboratory, Menlo Park, California 94025, USA

[5]*Advanced Photon Source, Argonne National Laboratory, Argonne, Illinois 60439, USA*

[6]*Department of Physics, University of California, Davis, California 95616, USA*

[7]*Department of Physics, Yale University, New Haven, Connecticut 06520, USA*

[8]*Department of Applied Physics, Yale University, New Haven, Connecticut 06520, USA*

[9]*Donostia International Physics Center, DIPC,*
20018 Donostia-San Sebastian, Basque Country, Spain

[10]*IKERBASQUE, Basque Foundation for Science, 48013 Bilbao, Spain*

[11]*Energy Sciences Institute, Yale University, West Haven, Connecticut 06516, USA*

[12]*Canadian Institute for Advanced Research,*
Toronto, Ontario ON M5G 1M1, Canada

---

* These authors contributed equally to this work.

† Present address: Department of Materials Science, Fudan University, 220 Handan Road, Shanghai, 200433, China

‡ afrano@ucsd.edu

Supplementary Methods

Reciprocal Space Dependence. The HL or KL-map data shown in Figure 2A of the main text were collected by taking a series of rocking curve scans with an incoming photon energy tuned to 932.4 eV. In this mode, the CCD detector angle (2θ) remains fixed while the sample angle (θ) is varied, scanning a trajectory within the KL-plane. A typical rocking curve trajectory is shown in Supplementary Figure 1 for the scan through the 3D CO center. Because the reciprocal space structure of the 3D CO is broad along H or K but narrow along L, a single rocking curve scan is effective at measuring the projection of the 3D CO peak along L, but requires a series of rocking curve scans to capture the projected width along H or K. Thus, rocking curve scans were measured for varying angles of the CCD detector (2θ), thereby spanning the entire region of the HL $orKL$-plane containing the 3D CO peak. Each CCD image was accumulated with an exposure time of 2 seconds per sample angle (θ). Each CCD pixel was converted to a reciprocal space index and the resultant three-dimensional scattering intensity data set was projected onto the HL or KL-plane.

The L-dependence of the (0 0 2) Bragg reflection shown in Figure 2C of the main text was obtained by taking a rocking curve (θ-scan) with 1746 eV photons. The data of the 3D CO stabilized in YBCO by magnetic field shown in Figure 2C of the main text was digitized from reference [1] (Figure 1J), which was measured with 98.5 keV x-rays at a temperature of 22 K under an applied 16.5 T magnetic field. The data of the 3D CO stabilized in YBCO by uniaxial strain shown in Figure 2C of the main text was digitized from reference [2] (Figure 3A), which was measured with 17.794 keV x-rays at a temperature of 50 K under an applied 1.0% uniaxial strain. The data of the 3D CO stabilized by epitaxial strain in YBCO thin films shown in Figure 2C of the main text was digitized from reference [3] (Figure 2G), which was measured at a temperature of 53 K with 932.7 eV x-rays tuned to the Cu L_3 resonance, representing the most directly comparable data to the present work, having been measured with very similar photon energies and scattering geometries, as well as being measured at very similar temperatures, due to both samples having a very similar superconducting T_c.

A further comparison between the 3D CO induced by epitaxial strain and the present work is shown in Supplementary Figure 2, which shows a plot similar to Figure 2 of the main text, but compares the 3D CO peak widths along the in-plane K reciprocal axis, instead of out-of-plane along L. The data shown in this figure was also digitized from reference [3] (Figure 1A) and was

also measured at a temperature of 53 K with 932.7 eV x-rays tuned to the Cu L_3 resonance.

No 2D CO rod signal was detected, being a central message of this work. It is still possible that a 2D CO phase exists but was too weak to be observed; for example, by being weakened due to surface contamination and/or from being fully oxygenated. Nonetheless, any undetected 2D CO would be orders of magnitude weaker than the observed 3D CO signal, which is in stark contrast to other reports where the 2D CO peak is weaker than, but of similar magnitude to, the 3D CO peak.

Energy Dependence. The x-ray absorption spectrum (XAS) was obtained at 50 K in total fluorescence yield mode using the CCD as a fluorescence detector, from which only the absorption edge peak locations were extracted as the data collected in TFY mode is likely not a good measure of the x-ray absorption coefficient, due to self-absorption corrections and the difference in quantum efficiency of the fluorescence yield for Pr M versus Cu L emissions. The energy resolution of incident x-rays for energies near the Cu and Pr resonance was $\sim$0.1 eV for this beam line.

The energy dependence of the 3D CO shown in Figure 3A of the main text was measured by performing a rocking curve scan for each energy at $T_c = 50K$ through the 3D CO peak maximum at (0 -0.335 1) and plotting the fitted areas, after subtracting the background contribution. This is preferable to measuring via an energy scan at fixed Q as the peak position can vary due to the energy dependence of the index of refraction. Because the reciprocal space structure of the 3D CO is broad along $H\ or\ K$ but narrow along L, a single rocking curve scan can effectively measure the projection of the peak along L.

The energy dependence of the full width at half maximum (FWHM) of the 3D CO peak is shown in Supplementary Figure 3 up to 935 eV, after which the fits become non-representative of the actual peaks due to decreased scattering signal. The FWHM of the 3D CO does not vary significantly over the energy range shown, likely due to the correlation length being limited by the coherence length of the crystal lattice.

Temperature Dependence. The temperature dependence of the 3D CO shown in Figure 3C of the main text was measured by performing a rocking curve scan for each temperature through the 3D CO peak maximum at (0 -0.335 1) and plotting the fitted areas, after subtracting background contributions. This was performed at two energies corresponding to the Pr M_5 and Cu L_3 absorption edges. Because the reciprocal space structure of the 3D CO is broad along $H\ or\ K$ but narrow along L, a single rocking curve scan can effectively measure the projection of the peak along L.

The temperature dependence of the FWHM of the 3D CO peak is shown in Supplementary

Figure 4, measured at the energies associated with peaks A and B in the 3D CO energy dependence, shown in Figure 3A of the main text. The FWHM of the 3D CO does not vary significantly over the temperature range shown, likely due to the correlation length being limited by the coherence length of the crystal lattice.

Density-Functional Calculations. We performed density-functional theory plus Hubbard U (DFT+U) calculations for both $PrBa_2Cu_3O_{6+\delta}$and $DyBa_2Cu_3O_{6+\delta}$ structures [4], where we chose the compounds without Y and for δ=0 in order to simplify the computational task without significantly impacting the underlying physics. $DyBa_2Cu_3O_{6+\delta}$ does not exhibit 3D CO, making the comparison against this compound more straightforward than against $YBa_2Cu_3O_{6+\delta}$, which displays both 2D and 3D CO. The calculations were done using the WIEN2K code [5–8] within the Perdew-Burke-Ernzerhof version of the generalized gradient approximation [9] on a 18 ×18 ×6 momentum grid. $R_{\mathrm{MT}}K_{\mathrm{max}}$ was set to 8.0 with muffin-tin radia R_{MT} = 2.50(2.48), 2.50, 1.87, and 1.61 a.u. for Pr(Dy), Ba, Cu, and O, respectively. To overcome the intrinsic shortcomings of DFT when dealing with open-shell core states, an effective Hubbard U parameter was imposed on the Pr/Dy $4f$ states. Considering that the Hartree-Fock values for the atomic Coulomb interactions are about 20% larger in Dy than those in Pr [10], we fixed $U_{\mathrm{eff}}^{\mathrm{Dy}} = 1.2U_{\mathrm{eff}}^{\mathrm{Pr}} = 6.0$ eV with $U_{\mathrm{eff}} = U - J$. We note that moderate variation of the U values as well as their ratio do not qualitatively change the results.

Supplementary Figure 5 shows the calculated band structure of $PrBa_2Cu_3O_6$ in the spin-majority channel in three dimensions. Apart from the $pd\sigma$ bands that are characteristic of the CuO_2 planes, an additional band crosses the Fermi level with dominantly Pr $4f$ and O $2p$ characters, which forms a cylindrical hole pocket around the SR line. Examination of the $4f$ density matrix shows that there are two occupied $4f$ states ($4f_{z(x^2-y^2)}$ and $4f_{z(5z^2-3)}$), indicating an electronic configuration of Pr^{3+}, in agreement with experimental observations [11, 12]. The $4f_{z(x^2-y^2)}$ state hybridizes with the eight nearest-neighbor O $2p_\pi$ states pointing towards Pr, resulting in the observed dispersive antibonding band with its top pushed above the Fermi level. It transfers holes from primarily planar O $2p_\sigma$ to $2p_\pi$ states [13, 14] and consequently, leads to strong electronic coupling between adjacent CuO_2 planes. The $4f_{z(5z^2-3)}$ state has only weak hybridization with the O $2p$ states and remains a flat energy level positioned around 1.1 eV below the Fermi level. In the spin-minority channel (not shown), on the other hand, the Pr $4f$ states remain empty and the top of the same band lies below the Fermi level at about -1.0 eV.

For heavier rare-earth elements with lower $4f$ energy, the $4f_{z(x^2-y^2)}$–$2p_\pi$ antibonding band is

expected to be lowered and removed from the Fermi level. Supplementary Figure 5B shows the spin-majority band structure for $DyBa_2Cu_3O_6$ in three dimensions for comparison.

For clarity, we also plot the two-dimensional projection of the bandstructure on the CuO_2 plane in Supplementary Figure 6, highlighting the 2 orbital characters of Pr/Dy $4f$ and planar O $2p$ are represented by purple and green, respectively. For Dy, the $4f$ is far below the Fermi level (not shown).

Supplementary Figure 1

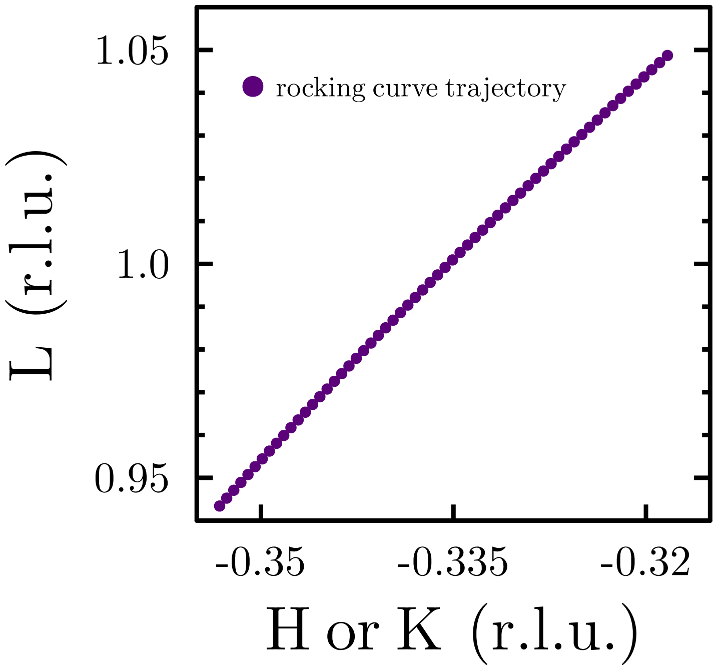

Supplementary Figure 1. **Rocking curve trajectory through the 3D CO.** The trajectory of a rocking curve scan through the location of the center of the three-dimensional charge order (3D CO) peak. In this mode, the CCD detector angle (2θ) remains fixed while the sample angle (θ) is varied. By measuring rocking curve scans through a range of CCD detector angles (2θ), various regions of the HL- or KL-plane may be spanned.

Supplementary Figure 2

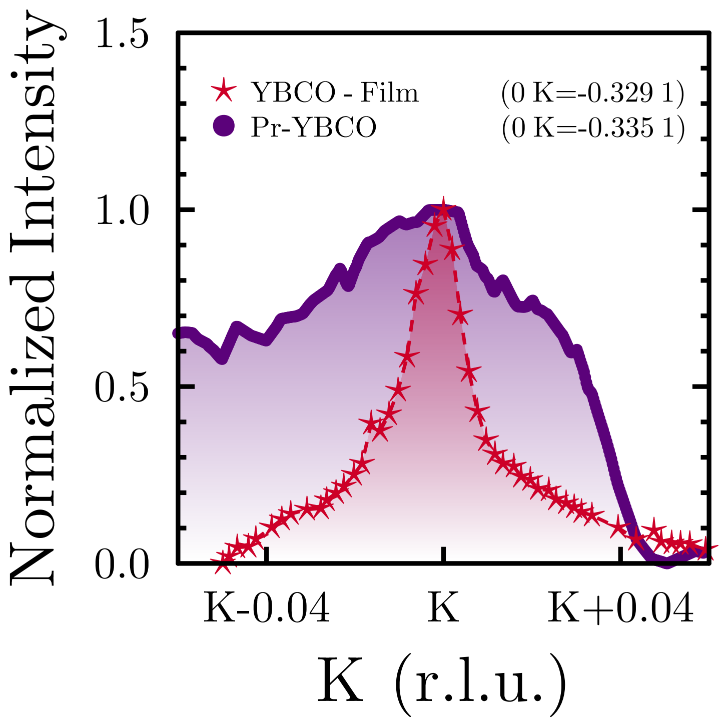

Supplementary Figure 2. **3D CO in-plane peak width comparison.** A comparison of the in-plane K-widths of the three-dimensional charge order (3D CO) peaks stabilized by epitaxial strain in YBCO films[3] and the present work.

Supplementary Figure 3

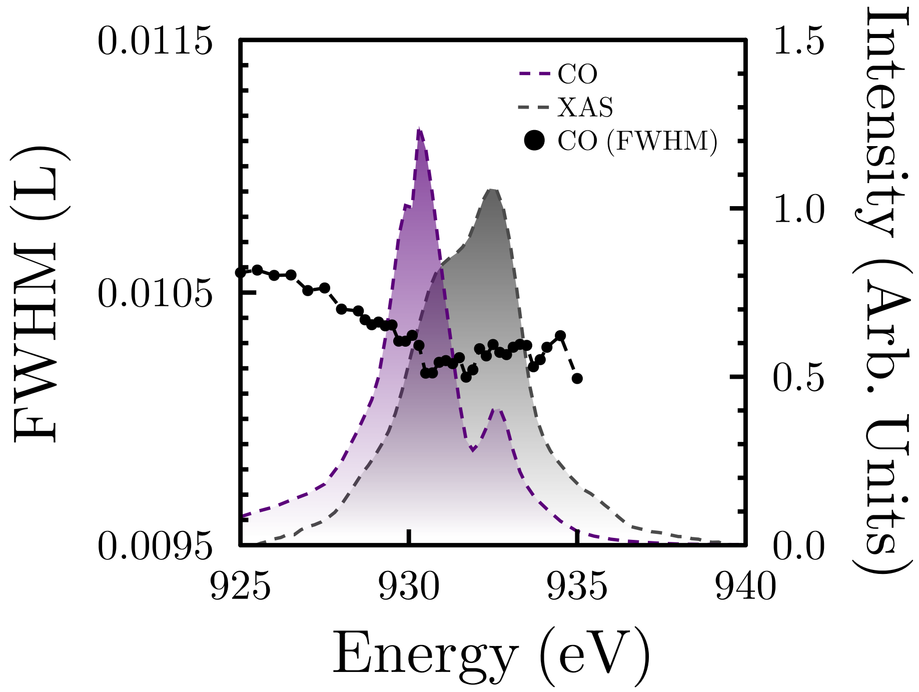

Supplementary Figure 3. **Energy dependence of the 3D CO peak width.** A plot showing the full width at half maximum (FWHM) (black), scattered intensity (purple), and x-ray absorption spectrum (gray) of the three-dimensional charge order (3D CO) at each energy.

Supplementary Figure 4

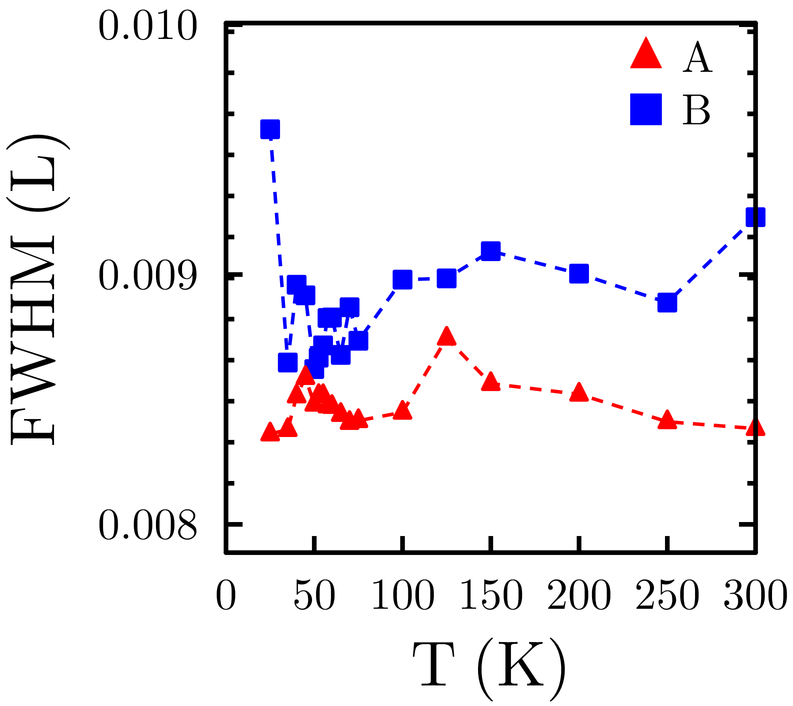

Supplementary Figure 4. **Temperature dependence of the 3D CO peak width.** A plot showing the full width at half maximum (FWHM) of the three-dimensional charge order (3D CO) peak at energies associated with the peaks A (red triangles) and B (blue squares) in the 3D CO energy dependence, shown in Figure 3A of the main text.

Supplementary Figure 5

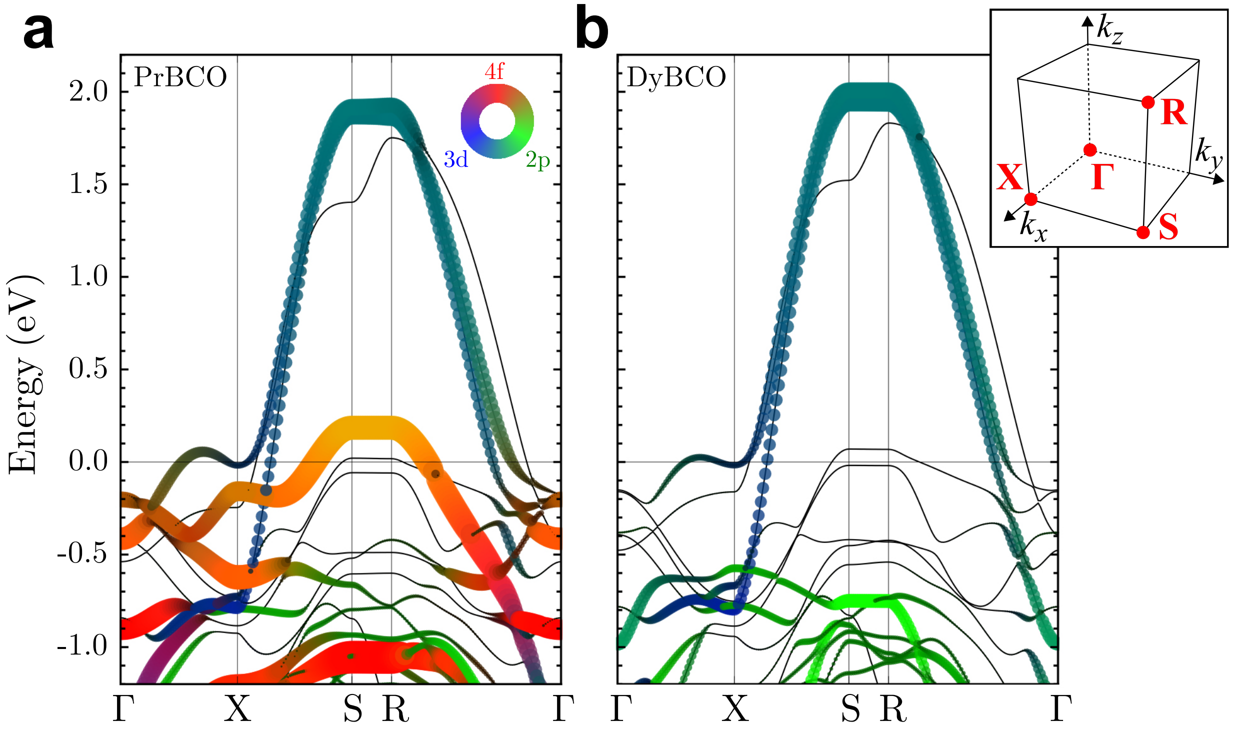

Supplementary Figure 5. **Spin-majority band structure in three dimensions.** Density-functional theory calculations of the three-dimensional band structure of **a** $PrBa_2Cu_3O_6$and **b** $DyBa_2Cu_3O_6$in the majority spin channel. The characters of Pr/Dy $4f$, planar Cu $3d_{x^2-y^2}$ and O $2p$ are represented by colors red, blue, and green, respectively. The line width is proportional to the combined characters above. In the $PrBa_2Cu_3O_6$ system, a band with mixed $4f$ and O $2p$ character crosses the Fermi level near the S and R points, while in the $DyBa_2Cu_3O_6$ system that band does not cross the Fermi level.

Supplementary Figure 6

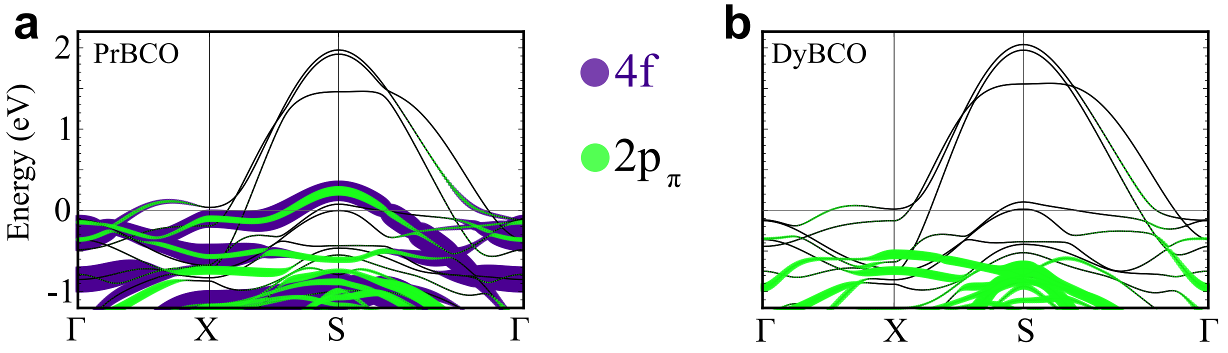

Supplementary Figure 6. **Projected bandstructure on the two-dimensional CuO_2 planes.** Planar density-functional theory plus Hubbard U (DFT+U) bandstructure of **a** $PrBa_2Cu_3O_6$and **b** $DyBa_2Cu_3O_6$. The characters of Pr/Dy $4f$, O $2p_\pi$ are represented by colors purple and green, respectively. The line width is proportional to the combined characters above and is not necessarily proportional to the density of states.

Supplementary Discussion

Identification of features in the 3D CO energy dependence. We turn to a brief discussion on the matter of identifying the features in the 3D CO energy dependence, which we believe to be warranted due to the close proximity of the Pr and Cu absorption edges. The XAS of the 3D CO shown in Figure 3A of the main text features two peaks at 930.9 eV and 932.6 eV, corresponding to the Pr M_5 and Cu L_3 absorption edges, respectively. The energy dependence of the 3D CO shown in Figure 3A of the main text also features two peaks at similar energies, which most likely correspond to the Pr and Cu resonances. Although the close proximity of these absorption edges prevents us from extracting the detailed spectroscopic information necessary to confirm the precise origin of the two features in the 3D CO energy dependence, we maintain a high level of confidence that they correspond to both Pr and Cu, as discussed below.

The most apparent support for this conclusion stems from the locations of the features in the energy dependence, which only deviate from the Pr M_5 and Cu L_3 absorption edge locations by -0.58 eV and 0.17 eV, respectively, which is consistent with prior resonant scattering studies of 3D CO in YBCO[3]. It seems sensible that the energy shift for the feature believed to be associated with the Pr M_5 edge is greater than for the feature believed to be associated with the Cu L_3 edge, considering that multi-peak structures in the energy dependence of rare-earth M edge scattering are common. Upon consideration of the absence of 3D CO in both pristine, unperturbed YBCO and in YBCO that has been substituted with rare earth elements other than Pr[15], combined with consideration of the absence of any similar, multi-peak structure in the energy dependence of 3D CO observed in YBCO induced by external perturbations[3, 16], it is conceivable that at least one feature in the energy dependence may be directly attributed to Pr atoms actively participating in the 3D CO formation. While it remains a technical possibility that both features in the energy dependence could be due to scattering from the Pr M_5 edge, with little to no participation from the Cu atoms, we believe this situation to be highly implausible due to the measured temperature dependence and known hybridization between Pr and the planar electronic states. As shown in Figure 3C of the main text, upon cooling, the 3D CO scattering exhibits a cusp-like maximum at the onset of superconductivity, with reduced scattering intensity for temperatures below T_c. This is the same trend that has been observed in the CO temperature dependence of all families of cuprates (in the absence of Pr) and has been attributed to a competition with superconductivity, which is believed to reside within the CuO_2 planes. It is challenging, then, to reconcile how the

hybridized Pr alone may exhibit a temperature dependence with the same distinct behavior as all other cuprates that do not contain Pr, which would presumably indicate a competition between the charge-ordered Pr atoms (located at the yttrium sites) and superconductivity in the CuO_2 planes, without any active participation by Cu. As such, we do not see any evidence of this in our data and believe the scenario to be highly unlikely. In culmination, while the energetic overlap between the Pr M_5 and Cu L_3 edges prevents precise determination of the origin of each feature in the 3D CO energy dependence, we believe that the simplest explanation of the observed phenomena is most likely — that the two features in the 3D CO energy dependence directly correspond to the Pr M_5 and Cu L_3 resonances observed in the XAS.

Supplementary Note

Secondary observation of 3D CO. A 3D CO signal was also observed and measured in a second sample of Pr-YBCO at SSRL BL 13-3 by the same methods as outlined above. The sample has a slightly higher T_c of 55 K, corresponding to a close, but slightly lower, Pr concentration. The crystalline quality is comparatively worse with less scattered intensity from Bragg reflections relative to the background and more pronounced twinning; as a result, the 3D CO scattered intensity is notably weaker. Due to some misalignment during the experimental setup, we refrain from drawing any quantitative conclusions (e.g., correlation lengths) from these measurements. Nonetheless, clear similarities between the characteristics of the 3D CO signal in these two samples can be drawn.

Similar to the primary sample, Supplementary Figure 7A displays that the XAS also resonates more strongly at the Cu L_3 edge, while the 3D CO also resonates more strongly at the featured believed to be associated with the Pr M_5 edge. Though, perhaps owing to the lower Pr concentration relative to the other sample, the peak ratios for both measurements are shifted in favor of the Cu L_3, which further suggests that the two features in the 3D CO energy dependence are associated with the Pr M_5 and Cu L_3 resonances observed in the absorption spectra. Supplementary Figure 7B and C show a reciprocal space structure that is broad along the $H\ or\ K$ axis and narrow along the L axis, respectively. Both observations are consistent with the characteristics of the 3D CO signal observed in the primary sample and confirm the existence of this phenomenon in this system. Measuring the 3D CO dependence on Pr concentration is a focus of ongoing work.

Supplementary Figure 7

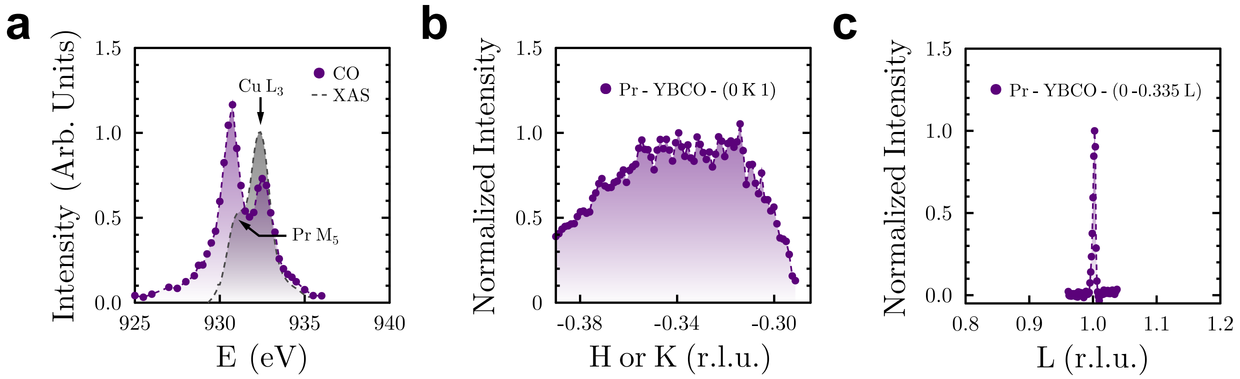

Supplementary Figure 7. **3D CO observed in a second sample of Pr-YBCO at T_c = 55 K. a** The x-ray absorption spectrum (XAS) (gray) resonates more strongly at the Cu L_3 edge, while the energy dependence of the three-dimensional charge order (3D CO) (purple) resonates more strongly at the feature believed to be associated with the Pr M_5 edge. The reciprocal space structure of the scattered 3D CO signal is **b** broad along the H or K axis and **c** narrow along the L axis. Both observations are consistent with the characteristics of the 3D CO observed in the primary sample.

Supplementary References

1. Chang, J., Blackburn, E., Ivashko, O., Holmes, A. T., Christensen, N. B., Hücker, M., Liang, R., Bonn, D. A., Hardy, W. N., Rütt, U., Zimmermann, M. V., Forgan, E. M., & Hayden, S. M. Magnetic field controlled charge density wave coupling in underdoped $YBa_2Cu_3O_{6+x}$. *Nat. Commun.* **7**, 11494 (2016).
2. Kim, H.-H., Souliou, S. M., Barber, M. E., Lefrançois, E., Minola, M., Tortora, M., Heid, R., Nandi, N., Borzi, R. A., Garbarino, G., Bosak, A., Porras, J., Loew, T., König, M., Moll, P. J. W., Mackenzie, A. P., Keimer, B., Hicks, C. W., & Le Tacon, M. Uniaxial pressure control of competing orders in a high-temperature superconductor. *Science* **362**, 1040–1044 (2018).
3. Bluschke, M., Frano, A., Schierle, E., Putzky, D., Ghorbani, F., Ortiz, R., Suzuki, H., Christiani, G., Logvenov, G., Weschke, E., Birgeneau, R. J., da Silva Neto, E. H., Minola, M., Blanco-Canosa, S., & Keimer, B. Stabilization of three-dimensional charge order in $YBa_2Cu_3O_{6+x}$. *Nat. Commun.* **9** (2018).
4. Guillaume, M., Allenspach, P., Mesot, J., Roessli, B., Staub, U., Fischer, P., & Furrer, A. A systematic neutron diffraction study of $R\mathrm{Ba_2Cu_3O_7}$ (R=yttrium and rare earths) high-T_c superconductors. *Z. Phys. B: Condens. Matter* **90**, 13–17 (1993).
5. Blaha, P., Schwarz, K., Madsen, G., Kvasnicka, D., Luitz, J., Laskowsk, R., Tran, F., & Marks, L. *WIEN2k: An Augmented Plane Wave Plus Local Orbitals Program for Calculating Crystal Properties*. (Techn. Universitat). (2019).
6. Blaha, P., Schwarz, K., Tran, F., Laskowski, R., Madsen, G. K. H., & Marks, L. D. WIEN2k: An APW+lo program for calculating the properties of solids. *J. Chem. Phys.* **152**, 074101 (2020).
7. Anisimov, V. I., Solovyev, I. V., Korotin, M. A., Czyżyk, M. T., & Sawatzky, G. A. Density-functional theory and NiO photoemission spectra. *Phys. Rev. B* **48**, 16929–16934 (1993).
8. Liechtenstein, A. I., Anisimov, V. I., & Zaanen, J. Density-functional theory and strong interactions: Orbital ordering in Mott-Hubbard insulators. *Phys. Rev. B* **52**, R5467–R5470 (1995).
9. Perdew, J. P., Burke, K., & Ernzerhof, M. Generalized gradient approximation made simple. *Phys. Rev. Lett.* **77**, 3865–3868 (1996).
10. Cowan, R. D. *The Theory of Atomic Structure and Spectra*. (Univ of California Press) No. 3. (1981).
11. Hilscher, G., Holland-Moritz, E., Holubar, T., Jostarndt, H.-D., Nekvasil, V., Schaudy, G., Walter, U., & Fillion, G. Valence of praseodymium in $Pr_xY_{1-x}Ba_2Cu_3O_{7-\delta}$: Inelastic-neutron-scattering, specific-heat, and susceptibility study. *Phys. Rev. B* **49**, 535–550 (1994).

12. Merz, M., Nücker, N., Pellegrin, E., Schweiss, P., Schuppler, S., Kielwein, M., Knupfer, M., Golden, M. S., Fink, J., Chen, C. T., Chakarian, V., Idzerda, Y. U., & Erb, A. X-ray absorption spectroscopy of detwinned $\mathrm{Pr_xY_{1-x}Ba_2Cu_3O_{7-y}}$ single crystals: Electronic structure and hole distribution. *Phys. Rev. B* **55**, 9160–9160 (1997).
13. Fehrenbacher, R. & Rice, T. M. Unusual electronic structure of $\mathrm{PrBa_2Cu_3O_7}$. *Phys. Rev. Lett.* **70**, 3471–3474 (1993).
14. Liechtenstein, A. I. & Mazin, I. I. Quantitative model for the superconductivity suppression in $R_{1-x}\mathrm{Pr}_x\mathrm{Ba_2Cu_3O_7}$ with different rare earths. *Phys. Rev. Lett.* **74**, 1000–1003 (1995).
15. Betto, D., Bluschke, M., Putzky, D., Schierle, E., Amorese, A., Fürsich, K., Blanco-Canosa, S., Christiani, G., Logvenov, G., Keimer, B., & Minola, M. Imprint of charge and oxygen orders on Dy ions in $\mathrm{DyBa_2Cu_3O}_{6+x}$ thin films probed by resonant x-ray scattering. *Phys. Rev. B* **102**, 195149 (2020).
16. Kim, H.-H., Lefrançois, E., Kummer, K., Fumagalli, R., Brookes, N. B., Betto, D., Nakata, S., Tortora, M., Porras, J., Loew, T., Barber, M. E., Braicovich, L., Mackenzie, A. P., Hicks, C. W., Keimer, B., Minola, M., & Le Tacon, M. Charge density waves in $\mathrm{YBa_2Cu_3O_{6.67}}$ probed by resonant x-ray scattering under uniaxial compression. *Phys. Rev. Lett.* **126**, 037002 (2021).
